# The 2019 WHO classification of tumours of the digestive system

**DOI:** 10.1111/his.13975

**Published:** 2019-11-13

**Authors:** Iris D Nagtegaal, Robert D Odze, David Klimstra, Valerie Paradis, Massimo Rugge, Peter Schirmacher, Kay M Washington, Fatima Carneiro, Ian A Cree, the WHO Classification of Tumours Editorial Board

**Affiliations:** ^1^ International Agency for Research on Cancer World Health Organization Lyon France

**Keywords:** cancer, classification, gastrointestinal tract, histopathology, molecular pathology

## Introduction

The *WHO classification of digestive system tumours* presented in the first volume of the *WHO classification of tumours series*, 5th edition, reflects important advancements in our understanding of tumours of the digestive system (Table 1). For the first time, certain tumour types are defined as much by their molecular phenotype as their histological characteristics; however, in most instances histopathological classification remains the gold standard for diagnosis. The *WHO classification of tumours* series is designed to be used worldwide, including those settings where a lack of tissue samples or of specific technical facilities limits the pathologist's ability to rely on molecular testing.

**Table 1:** Selected changes within the new classification of tumours of the digestive system

Type	Subject	Change in 2019 classification
Oesophageal adenocarcinoma	Aetiology and epidemiology	The epidemiology has been updated: 7% of cases are thought to be familial, and the risk factors involved in sporadic cases have been updated. The role of gastro‐oesophageal reflux in the inflammation–metaplasia–dysplasia adenocarcinoma model has been emphasised
Oesophageal adenocarcinoma	Prognosis and prediction	The use of antibodies targeting ERBB2 (HER2) in patients overexpressing this molecule is included, and the need for testing
Oesophageal squamous carcinoma and oesophageal squamous dysplasia	Aetiology and pathogenesis	The potential role of HPV remains uncertain. Other environmental factors, including tobacco and alcohol consumption appear to be more important. The importance of *TP53* mutation is now clear, and studies have identified alterations in genes that regulate cell cycle, cell differentiation (especially NOTCH pathway) and EGFR (HER1) signalling as key genetic abnormalities
Gastric adenocarcinoma	Aetiology and pathogenesis	Most sporadic gastric cancers are now considered to be inflammation‐driven, and their aetiology is characteristically environmental – usually related to *Helicobacter pylori* infection. Up to 10% of gastric cancers are familial. Other factors include tobacco smoking, irradiation and diet. Molecular subtypes as proposed by two consortia are described, although clinical application is limited
Gastric adenocarcinoma	Classification	Heterogeneity of poorly cohesive carcinoma (PCC) is discussed, including signet‐ring cell carcinoma and PCC‐NOS. Rare subtypes are described, such as gastric adenocarcinoma of fundic‐gland type
Gastric adenocarcinoma	Prognosis and prediction	ERBB2 testing is used to predict potential response to anti‐ERBB2 therapy. MSI‐H and EBV positivity are markers of good prognosis with potential therapeutic importance, namely for immunotherapy targeting the PD‐1/PD‐L1 axis (under investigation in clinical trials). A large number of other reported markers are described, but not yet in practice
Small intestinal and ampullary carcinomas	Pathogenesis	These are split into ampullary and non‐ampullary types, on the basis of anatomy. Pathogenesis seems similar to colorectal carcinoma, though more information is required
Goblet cell adenocarcinoma of the appendix	Classification	This is a change from goblet cell carcinoid/carcinoma as it is now recognised to have a minor neuroendocrine component
Serrated lesions of the colon, rectum and appendix	Classification and pathogenesis	The preferred name is serrated lesion, as these may be flat rather than polypoid, and the association with *BRAF* or *KRAS* mutation delineates two separate neoplastic pathways
Anal squamous dysplasia	Diagnostic molecular pathology	P16 and HPV testing is recommended
Neuroendocrine neoplasms (NEN)	Classification and molecular pathology	The general principles of the new classification of neuroendocrine tumours (NET) will be applied to the entire 5th series, based on a consensus meeting in Lyon (1), dividing NEN into NET and neuroendocrine carcinomas (NEC) based on their molecular differences. Mutations in MEN1, DAXX and ATRX are entity‐defining for well‐differentiated NETs, while NECs usually have *TP53* or *RB1* mutations
Precursor lesions	Classification	The term ‘dysplasia’ is preferred for lesions in the tubal gut, whereas ‘intra‐epithelial neoplasia’ is preferred for those in the pancreas, gallbladder and biliary tree. Use of the term ‘carcinoma *in situ*’ is not recommended
Hepatocellular tumours	Classification	Revision based on molecular profiling studies. Fibrolamellar carcinoma defined by *DNAJB1*–*PRKACA* translocation
Intrahepatic cholangiocarcinoma	Classification	Two main subtypes: a large duct type, which resembles extrahepatic cholangiocarcinoma, and a small duct type, which shares aetiological, pathogenetic and imaging characteristics with hepatocellular carcinoma
Pancreatic intraductal neoplasms	Classification	Intraductal oncocytic papillary and intraductal tubulopapillary neoplasms are distinguished from intraductal papillary mucinous neoplasms and ductal adenocarcinoma by the absence of *KRAS* in these lesions
Acinar cystic transformation of the pancreas	Classification	Previously called acinar cell cystadenoma, but now demonstrated to be non‐neoplastic by molecular clonality analysis
Haematolymphoid tumours and mesenchymal tumours	Classification	Grouped together in separate chapters, to ensure consistency and avoid duplication
EBV‐positive inflammatory follicular dendritic cell sarcoma of the digestive tract	Classification	This name change is necessary due to new information on the EBV relationship of this tumour type, previously known as ‘inflammatory pseudotumour‐like fibroblastic/follicular dendritic cell tumour’
Genetic tumour syndromes of the digestive system	Classification, pathogenesis and diagnostic molecular pathology	Common syndromes are updated. A new section on GAPPS (gastric adenocarcinoma and proximal polyposis of the stomach) syndrome is presented. Tumour predisposition syndromes that confer a raised risk of various gastrointestinal tumours are described

EBV, Epstein–Barr virus; HPV, Human papillomavirus; PD‐1, Programmed death 1; PD‐L1, Programmed death ligand; NOS, Not otherwise specified; EGFR, Epidermal growth factor receptor; HER1, Human epidermal growth factor receptor 1.

Rindi *et al.* 3

Since the publication of the 4th‐edition digestive system tumours volume in 2010,^1^ there have been important developments in our understanding of the aetiology and pathogenesis of many tumours. However, the extent to which this new information has altered clinical practice has been quite variable. For some of the tumours described in this volume there is little molecular pathology in clinical use, despite the fact that we now have a more detailed understanding of their molecular pathogenesis. A tumour's molecular pathology, as defined for the purposes of this publication, concerns the molecular markers that are relevant to the tumour's diagnosis, biological behaviour, outcome and treatment, rather than its molecular pathogenesis. However, the role of molecular pathology is expanding; for some tumour entities, molecular analysis is now essential for establishing an accurate diagnosis. Some of these analyses require investigation of somatic (acquired) genetic alterations, gene or protein expression, or even circulating tumour markers. For certain tumour types, specific analytical tests are needed to predict prognosis or tumour progression, and these tests are carefully outlined in this volume. In the following paragraphs, we have summarised some of the more notable changes since the 4th edition. In instances where the new WHO classification of tumours editorial board determined that there was insufficient evidence of the diagnostic or clinical relevance of new information about a particular tumour entity, the position held in the 4th edition has been maintained as the standard in the new volume.

## Oesophageal and gastric tumours

There has been substantial progress in our understanding of the development of glandular oesophageal neoplasia and the sequential neoplastic progression from inflammation to metaplasia (Barrett's oesophagus), dysplasia and, ultimately, adenocarcinoma. This process is initially driven by gastro‐oesophageal reflux disease, which leads to reprogramming of cell differentiation and proliferation in the oesophagus. There is evidence that *TP53* mutation in proliferating epithelium leads to high‐grade dysplasia, while *SMAD4* mutation precedes the development of invasive carcinoma. While demonstration of these mutations is not required clinically, testing oesophageal and gastric adenocarcinomas for ERBB2 [human epidermal growth factor receptor 2 (HER2)] is recommended, as this influences treatment decisions.

The pathogenesis of precursor lesions is less clear in oesophageal squamous carcinogenesis than in gastric carcinogenesis. Environmental factors are believed to play an important role, but the mechanisms of neoplastic change as a result of specific factors, such as tobacco use and alcohol consumption, are poorly understood. For example, human papillomavirus (HPV) infection was initially believed to play a key role in squamous carcinogenesis, but recent evidence suggests that there is no such association in most cases of oesophageal squamous cell carcinoma.

The molecular pathway of cancer progression in the stomach is less clear. Most epidemic gastric cancers are now considered inflammation‐driven, and their aetiology is characteristically environmental – usually related to *Helicobacter pylori* infection. It is because of this infectious aetiology that gastric cancer is included among the limited number of highly lethal, but preventable, cancers. Chronic gastric inflammation leads to changes in the microenvironment (including the microbiome) that results in mucosal atrophy/metaplasia, which may then progress to neoplasia after further molecular alterations. Metaplastic changes in the upper gastrointestinal tract are well‐recognised as early cancer precursors, but their precise molecular mechanisms and the exact role of progenitor cells in the oncogenic cascade remain a subject of intense investigation. For some rare tumours, distinctive driver mutations have been identified; for example, the characteristic *MALAT1*–*GLI1* fusion gene in gastroblastoma and *EWSR1* fusions in gastrointestinal clear cell sarcoma and malignant gastrointestinal neuroectodermal tumour. In both examples, demonstration of the fusion gene is now required for the diagnosis.

## Tumours of the anus, small and large intestines

The pathogenesis of adenocarcinomas of the intestines (the small and large bowel and the appendix) is now much better delineated than it was a decade ago. The introduction of population‐based screening for colorectal cancer has laid the foundation for a better understanding of neoplastic precursor lesions and the molecular pathways associated with each type of tumour. For example, our knowledge of the molecular pathways and biological behaviour of conventional adenomas and serrated precursor lesions, including the recently renamed sessile serrated lesion (formerly called sessile serrated polyp/adenoma), has grown rapidly in the past decade, and this has enabled clinicians to provide tailored, evidence‐driven screening and surveillance programmes. Colorectal cancers, in which it will make a difference to patient treatment, should undergo molecular testing for microsatellite instability and extended RAS testing for mutations in *KRAS*, *NRAS* and *BRAF*. Our understanding of appendiceal tumours has also improved. For example, we now know that many tumours of the appendix develop via neoplastic precursor lesions similar to those in the small and large intestines, and the biological potential and molecular pathways of appendiceal tumours are therefore much better appreciated. The recently renamed goblet cell adenocarcinoma (formerly called goblet cell carcinoid/carcinoma) of the appendix is a prime example of a tumour whose biological potential and histological characteristics have been better described, resulting in improvements in the pathological approach to these tumours. Studies of the aetiology and pathogenesis of anal squamous lesions suggests that HPV infection plays an important aetiological role, driving genetic alterations similar to those in cervical cancer. p16 and HPV testing are recommended for such lesions.

## Neuroendocrine neoplasms

One particularly important change in the 5th edition is in the classification of neuroendocrine neoplasms (NENs), which occur in multiple sites throughout the body. In this volume, NENs are covered within each organ‐specific chapter, including the chapter on tumours of the pancreas, where detailed sections describing each functioning and non‐functioning subtype are provided. Previously, these neoplasms were covered only in the volume on tumours of endocrine organs.^2^ The general principles guiding the classification of all NENs are presented in a separate introduction to this topic (Table 2). To consolidate our increased understanding of the genetics of these neoplasms, a group of experts met for a consensus conference at the International Agency for Research on Cancer (IARC) in November 2017 and subsequently published a paper in which they proposed distinguishing between well‐differentiated neuroendocrine tumours (NETs) and poorly differentiated neuroendocrine carcinomas (NECs) in all sites where these neoplasms arise.^3^ NEN are divided into NET and NECs, based on their molecular differences. Mutations in *MEN1*, *DAXX* and *ATRX* are entity‐defining for well‐differentiated NETs, whereas NECs usually have *TP53* or *RB1* mutations. In some cases, these mutations can be of diagnostic benefit. Genomic data have also led to a change in the classification of mixed NENs, which are now grouped into the conceptual category of ‘mixed neuroendocrine–non‐neuroendocrine neoplasms (MiNENs)’. Mixed adenoneuroendocrine carcinomas (MANECs), which show genomic alterations similar to those of adenocarcinomas or NECs rather than NETs, probably reflect clonal evolution within the tumours, which is a rapidly growing area of interest. The study of these mixed carcinomas may also lead to an improved understanding of other facets of clonality in tumours of the digestive system and other parts of the body.

**Table 2:** Classification and grading criteria for neuroendocrine neoplasms (NENs) of the GI tract and hepatopancreatobiliary organs

Terminology	Differentiation	Grade	Mitotic rate* (mitoses/2 mm^2^)	Ki‐67 index*
NET, G1	Well differentiated	Low	<2	<3%
NET, G2		Intermediate	2–20	3–20%
NET, G3		High	>20	>20%
NEC, small‐cell type (SCNEC)	Poorly differentiated	High†	>20	>20%
NEC, large‐cell type (LCNEC)			>20	>20%
MiNEN	Well or poorly differentiated‡	Variable‡	Variable‡	Variable‡

LCNEC, Large‐cell neuroendocrine carcinoma; MiNEN, Mixed neuroendocrine–non‐neuroendocrine neoplasm; NEC, Neuroendocrine carcinoma; NET, Neuroendocrine tumour; SCNEC, Small‐cell neuroendocrine carcinoma.

* Mitotic rates are to be expressed as the number of mitoses/2 mm^2^ as determined by counting in 50 fields of 0.2 mm^2^ (i.e. in a total area of 10 mm^2^); the Ki‐67 proliferation index value is determined by counting at least 500 cells in the regions of highest labelling (hot‐spots), which are identified at scanning magnification; the final grade is based on whichever of the two proliferation indexes places the neoplasm in the higher‐grade category.

^†^ Poorly differentiated NECs are not formally graded, but are considered high‐grade by definition.

^‡^ In most MiNENs, both the neuroendocrine and non‐neuroendocrine components are poorly differentiated, and the neuroendocrine component has proliferation indices in the same range as other NECs, but this conceptual category allows for the possibility that one or both components may be well differentiated; when feasible, each component should therefore be graded separately.

Another important change concerns the recognition that well‐differentiated NETs may be high grade (G3 in the WHO grading system, defined as having a mitotic rate >20 per 2 mm^2^ or Ki67 >20%), but these neoplasms remain well‐differentiated genetically and distinct from poorly differentiated NECs. G3 NETs were first recognised and are most common in the pancreas, but they can occur throughout the GI tract. Thus, the current WHO classification includes three grades (G1, G2 and G3) for NETs. NECs are no longer graded, as they are recognised to be uniformly high grade by definition, but continue to be separated into small‐and large‐cell types.

## Precursor lesions

There are certain terms in current day‐to‐day use about which many pathologists continue to disagree. The editorial board carefully considered our current understanding of carcinogenetic pathways when considering the use of specific terms and definitions. In general, the overall consensus was that established terms, definitions and criteria should not be changed unless there was strong evidence to support doing so and the proposed changes had clinical relevance. For some tumours, our understanding of the progression from normal epithelium to metastatic carcinoma remains inadequate. For example, in certain tumours the line between benign and malignant can be ambiguous, and in some cases the distinction is more definitional than biological. These are some of the many areas of tumour biology that need to be more fully investigated in the future.

In the 5th edition, the terminology for precursors to invasive carcinoma in the digestive system has been standardised somewhat, although the terms ‘dysplasia’ and ‘intra‐epithelial neoplasia’ are both still considered acceptable for lesions in certain anatomical locations, in acknowledgement of their ongoing clinical acceptance. For example, the term ‘dysplasia’ is preferred for lesions in the tubular gut, whereas ‘intra‐epithelial neoplasia’ is preferred for those in the pancreas, gallbladder and biliary tree. For all anatomical sites, however, a two‐tiered system (low‐ versus high‐grade) is considered the standard grading system for neoplastic precursor lesions. This has replaced the three‐tiered grading scheme previously used for lesions in the pancreatobiliary system.^4^ The term ‘carcinoma *in situ*’ continues to be strongly discouraged in clinical practice for a variety of reasons, most notably its clinical ambiguity. This term is encompassed by the category of high‐grade dysplasia/intraepithelial neoplasia.

## Liver tumours

Many refinements of the 4th‐edition classification have been made concerning liver tumours, supported by novel molecular findings. For example, a comprehensive picture of the molecular changes that occur in common hepatocellular carcinoma has recently emerged from large‐scale molecular profiling studies. Meanwhile, several rarer hepatocellular carcinoma subtypes, which together may account for 20–30% of cases, have been defined by consistent morphomolecular and clinical features, with fibrolamellar carcinoma and its diagnostic *DNAJB1*–*PRKACA* translocation being one prime example. Intrahepatic cholangiocarcinoma is now understood to be an anatomically defined entity with two different major subtypes: a large duct type, which resembles extrahepatic cholangiocarcinoma, and a small duct type, which shares significant aetiological, pathogenetic and imaging characteristics with hepatocellular carcinoma. The two subtypes have very different aetiologies, molecular alterations, growth patterns and clinical behaviours, exemplifying the conflict between anatomically and histogenetically/pathogenetically based classifications. Clinical research and study protocols will need to incorporate these findings in the near future. Also supported by molecular findings, the definition of combined hepatocellular–cholangiocarcinoma and its distinction from other entities has recently become clearer. Cholangiolocellular carcinoma is no longer considered a subtype of combined hepatocellular–cholangiocarcinoma, but rather a subtype of small duct intrahepatic cholangiocarcinoma, renamed cholangiolocarcinoma, meaning that all intrahepatic carcinomas with a ductal or tubular phenotype are now included within the category of intrahepatic cholangiocarcinoma. A classic example of morphology‐based molecular profiling leading to a new classification based on a combination of biological and molecular factors is the classification of hepatocellular adenomas, which has gained a high degree of clinical relevance and has fuelled the implementation of refined morphological criteria and molecular testing in routine diagnostics.

## Tumours of the pancreas

Most of the classification of pancreatic neoplasms in the 5th edition remains unchanged from the last volume. As highlighted above, precursor lesions including pancreatic intraepithelial neoplasia, intraductal papillary mucinous neoplasms and mucinous cystic neoplasms are now classified into two tiers of dysplasia, based on the highest grade of dysplasia detected, rather than the three‐tier system used in the last edition of the WHO classification. Intraductal oncocytic papillary neoplasm and intraductal tubulopapillary neoplasms are now separated from the other subtypes of intraductal papillary mucinous neoplasm based on their distinct genomic and morphological features. The prior entity of acinar cell cystadenoma, which has recently been demonstrated to be non‐neoplastic by molecular clonality analysis, is now termed ‘acinar cystic transformation of the pancreas’. Also, the entire spectrum of pancreatic neuroendocrine neoplasms is now included in this volume; previously, details concerning the individual functional types were presented in the *WHO classification of tumours of the endocrine organs*.

## Mixed tumours

Mixed tumours in several anatomical sites (e.g. oesophageal adenosquamous carcinoma and mucoepidermoid carcinoma, as well as hepatic carcinomas with mixed hepatocellular and cholangiocellular differentiation), remain subjects of some uncertainty. The relative importance of the various lineages of differentiation within these neoplasms remains unknown. It is also uncertain how these neoplasms develop and how they should be treated. These issues are a matter of debate because hard evidence is lacking, but there are improvements in the pathological criteria and classification of these neoplasms that should help to standardise the diagnostic approach and facilitate better clinical and genomic research.

## Haematolymphoid tumours and mesenchymal tumours

Each of these tumour types is grouped together in separate chapters. This ensures consistency and avoids duplication. The term ‘EBV positive inflammatory follicular dendritic cell sarcoma of the digestive tract’ has been adopted to replace the entity previously known as ‘inflammatory pseudotumour‐like fibroblastic/follicular dendritic cell tumour’.

## Genetic tumour syndromes

New in this book is the chapter on genetic tumour syndromes of the digestive system, the introduction to which contains a table that lists each of the major syndromes and summarises key information about the disease/phenotype, pattern of inheritance, causative gene(s) and normal function of the encoded protein(s). Common syndromes, including Lynch syndrome and familial adenomatous polyposis 1 (FAP), are covered in detail, as well as several other adenomatous polyposes defined since the last volume and the GAPPS (gastric adenocarcinoma and proximal polyposis of the stomach) syndrome, now recognised as a FAP variant, with a unique phenotype. A number of other genetic tumour predisposition syndromes that confer a raised risk of various gastrointestinal tumours are also described, including Li–Fraumeni syndrome, hereditary haemorrhagic telangiectasia, syndromes associated with gastroenteropancreatic NETs and multilocus inherited neoplasia alleles syndrome. This should be helpful to many involved in the diagnosis of such syndromes, as well as those researching the mechanisms involved.

## Format changes

The format of the books has been updated to reflect the new edition of the classification: the move from three to two columns has allowed larger illustrations, and the use of set headings for each tumour type show very clearly where evidence is lacking.

## Conflict of interest

I.D.N. reports that her institute benefits from research funding from the Dutch Cancer Society (KWF) and the Dutch Digestive Foundation (MLDS). No other authors report any conflicts of interest to IARC that would affect their participation in forming the classification.
